# Supplementary material for: Peripheral modulation of antidepressant targets MAO-B and GABAAR by harmol induces mitohormesis and delays aging in preclinical models
Source: Nat Commun. 2023 May 15;14:2779. doi: 10.1038/s41467-023-38410-y (PMC10185515; doi:10.1038/s41467-023-38410-y)
Supplement: Supplementary file 1 — Supplementary Information [file 41467_2023_38410_MOESM1_ESM.pdf]

Supplementary Files for:

# Peripheral modulation of antidepressant targets MAO-B and GABAAR by harmol induces mitohormesis and delays aging in preclinical models

Luis Filipe Costa-Machado[1,2,3], Esther Garcia-Dominguez[4], Rebecca L. McIntyre[5], Jose Luis Lopez-Aceituno[1], Álvaro Ballesteros-Gonzalez[6], Andrea Tapia-Gonzalez[7], David Fabregat-Safont[8,9], Tobias Eisenberg[10,11,12], Jesús Gomez[13], Adrian Plaza[1], Aranzazu Sierra-Ramirez[1], Manuel Perez[13], David Villanueva-Bermejo[14], Tiziana Fornari[14], María Isabel Loza[2,3], Gonzalo Herradon[15], Sebastian J. Hofer[10,11,12], Christoph Magnes[16], Frank Madeo[10,11,12], Janet S. Duerr[17], Oscar J. Pozo[8], Maximo-Ibo Galindo[6,18,19], Isabel del Pino[7,20], Riekelt H. Houtkooper[5], Diego Megias[13], Jose Viña[4], Mari Carmen Gomez-Cabrera[4], Pablo J Fernandez-Marcos[1,*].

1 Metabolic Syndrome Group – BIOPROMET. Madrid Institute for Advanced Studies - IMDEA Food, CEI UAM+CSIC, E28049, Madrid Spain.
2 Fundación Kærtor, Edificio EMPRENDIA, Planta 2, Oficina 4. Campus Vida, E-15706 Santiago de Compostela, Spain.
3 BioFarma, Center for Research in Molecular Medicine and Chronic Diseases (CIMUS), Universidade de Santiago de Compostela, Health Research Institute of Santiago de Compostela (IDIS), Santiago de Compostela, Spain.
4 Freshage Research Group, Department of Physiology, Faculty of Medicine, CIBERFES, Fundación Investigación Hospital Clínico Universitario/INCLIVA, University of Valencia, Valencia, Spain.
5 Laboratory Genetic Metabolic Diseases, Amsterdam Gastroenterology, Endocrinology, Metabolism, Amsterdam Cardiovascular Sciences, Amsterdam UMC, University of Amsterdam, Amsterdam, Netherlands.
6 Developmental Biology and Disease Models Group, Centro de Investigación Príncipe Felipe, 46012 Valencia, Spain.

7 Neural Plasticity Group, Centro de Investigación Príncipe Felipe, 46012 Valencia, Spain.
8 Applied Metabolomics Research Group, Hospital del Mar Medical Research Institute - (IMIM), Barcelona, Spain.
9 Environmental and Public Health Analytical Chemistry, Research Institute for Pesticides and Water, University Jaume I, 12006 Castellón, Spain.
10 Institute of Molecular Biosciences, NAWI Graz, University of Graz, Humboldtstraße 50, 8010, Graz, Austria.
11 BioTechMed Graz, 8010, Graz, Austria.
12 Field of Excellence BioHealth – University of Graz, Austria.
13 Confocal Microscopy Unit, Biotechnology Programme, Spanish National Cancer Research Centre (CNIO), Melchor Fernández Almagro 3, 28029 Madrid, Spain.
14 Department of Production and Characterization of Novel Foods, Institute of Food Science Research (CIAL UAM-CSIC), C/ Nicolás Cabrera, 9, P.O. Box. 28049, Madrid, Spain.
15 Lab. Pharmacology, Faculty of Pharmacy, Universidad CEU San Pablo, Urb. Montepríncipe, 28668 Boadilla del Monte, Madrid, Spain.
16 HEALTH-Institute for Biomedicine and Health Sciences, Joanneum Research Forschungsgesellschaft mbH, 8010 Graz, Austria.
17 Department of Biological Sciences, Ohio University, Athens, OH 45701, USA.
18 Instituto Interuniversitario de Investigación de Reconocimiento Molecular y Desarrollo Tecnológico (IDM), Universitat Politècnica de València, Universitat de València. 46022 Valencia, Spain.
19 UPV-CIPF Joint Research Unit “Disease Mechanisms and Nanomedicine”. Centro de Investigación Príncipe Felipe, 46012 Valencia, Spain.
20 Instituto de Neurociencias, Consejo Superior de Investigaciones Científicas, Universidad Miguel Hernández, Campus de Sant Joan, 03550 Alicante, Spain.

**Short title: Harmol induces mitohormesis and improves healthspan**

* *Materials and correspondence should be requested to:*
pablojose.fernandez@imdea.org

**Index:**

- *Supplementary Figures 1-7*, each one complementing the information shown in its corresponding Main Figure.
- *Supplementary Tables 1 and 2*, complementing Figure 4a-b.
- *Supplementary Data 1*, complementing Figure 1.
- *Supplementary Data 2*, complementing Figure 3c and 5r.
- *Supplementary Data 3*, complementing Figure 4a-b.
- *Supplementary Data 4*, complementing Figure 6.

# Figure S1

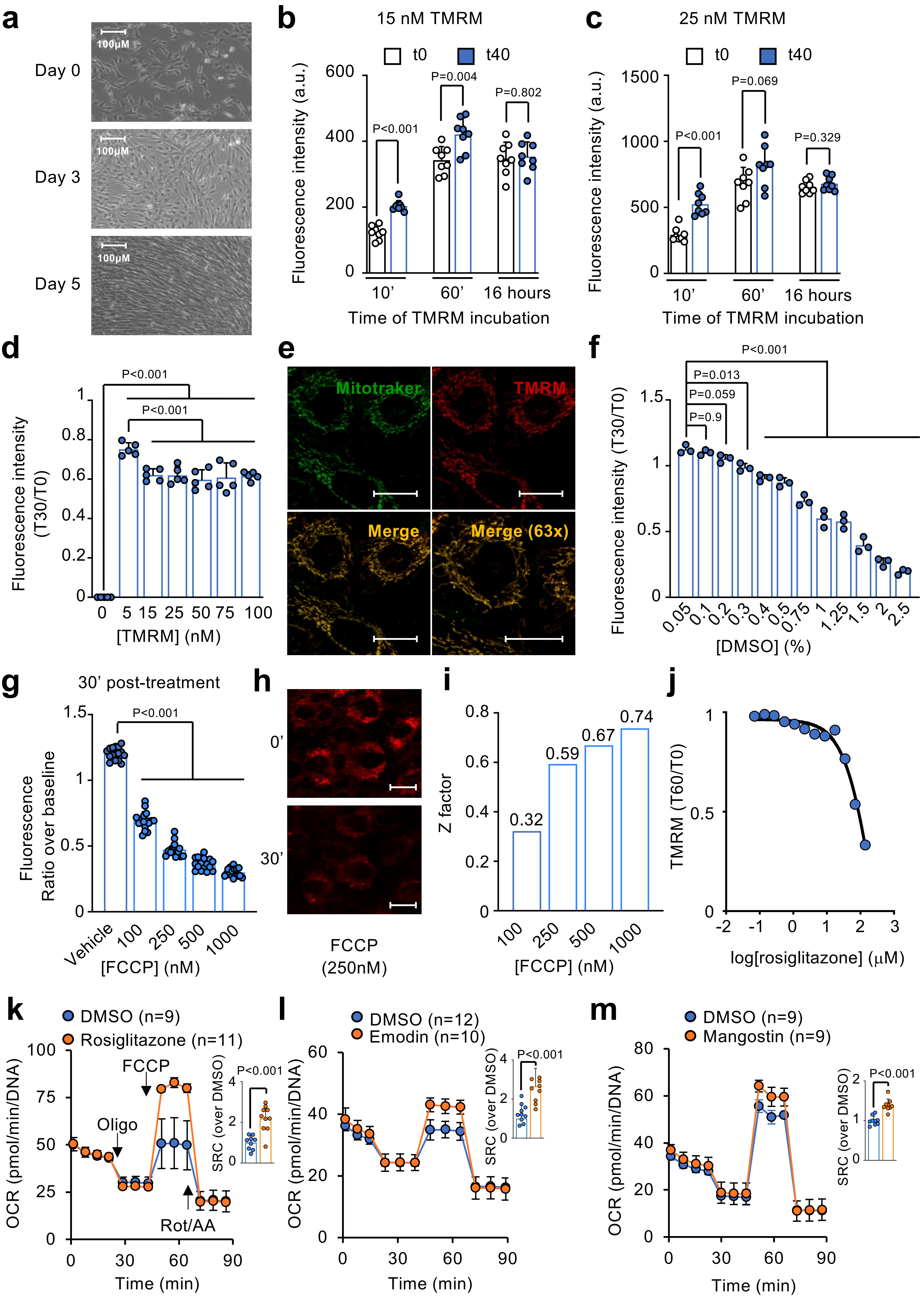
a
Day 0
Day 3
Day 5
100μM
b
15 nM TMRM
t0
t40
P<0.001
P=0.004
P=0.802
Fluorescence intensity (a.u.)
10'
60'
16 hours
Time of TMRM incubation
c
25 nM TMRM
P<0.001
P=0.069
P=0.329
d
Fluorescence intensity (T30/T0)
[TMRM] (nM)
e
Mitotraker
TMRM
Merge
Merge (63x)
f
P<0.001
P=0.013
P=0.059
P=0.9
[DMSO] (%)
g
30' post-treatment
Fluorescence Ratio over baseline
Vehicle
[FCCP] (nM)
h
0'
30'
FCCP (250nM)
i
Z factor
0.32
0.59
0.67
0.74
[FCCP] (nM)
j
TMRM (T60/T0)
log[rosiglitazone] (μM)
k
DMSO (n=9)
Rosiglitazone (n=11)
FCCP
Oligo
Rot/AA
OCR (pmol/min/DNA)
SRC (over DMSO)
Time (min)
l
DMSO (n=12)
Emodin (n=10)
m
DMSO (n=9)
Mangostin (n=9)

**Supplementary Figure S1. (a)** Phase contrast microscopy images of C2C12 myoblast cells cultured in differentiation medium for the indicated times to induce an efficient differentiation into myotubes. **(b, c)** TMRM fluorescent intensity obtained after treatment with 15 nM (b) or 25 nM (c) TMRM during the times indicated in the X axis, measured at one given time (t0) and 40 minutes after t0 (t40). **(d)** Fluorescence intensity of the indicated TMRM concentrations expressed as the ratio between baseline without treatment (T0) and 30 minutes (T30) after addition of 100nM FCCP. **(e)** Confocal images of total mitochondria (Mitotracker, in green) and TMRM (in red) of differentiated C2C12 myotubes treated with 15 nM TMRM for 16 hours. Size bar=20 μM. **(f)** TMRM fluorescent intensity after treatment with the indicated concentrations of DMSO. **(g)** TMRM fluorescent intensity expressed as the ratio between baseline without treatment (T0) and 30 minutes after treatment (T30) with the indicated concentrations of FCCP. **(h)** Confocal images of TMRM (in red) of differentiated C2C12 cells prior to FCCP treatment (top panel) and 30' after treatment with 250 nM FCCP (bottom panel). Size bar=20 μM. **(i)** Z factor score with 16 replicates between baseline (0.1% DMSO) and 30 minutes after the addition of the indicated concentrations of FCCP. **(j)** C2C12 myotubes stained with TMRM were treated with the indicated concentrations of rosiglitazone, and TMRM fluorescence intensity was recorded before (T0) and 30 minutes after (T30) treatment with rosiglitazone. **(k-m)** Oxygen consumption rates (OCR) in differentiated C2C12 myotubes treated for 16 hours with 30 μM of rosiglitazone (j), 1.3 μg/ml emodin (k) or 1.3 μg/ml mangostin (l) following the sequential addition of oligomycin A (Oligo), FCCP and Rotenone/Antimycin A (Rot/AA) at the indicated times (Mitostress Seahorse experiment). Small panels on the right represent the spare respiratory capacity parameter of the complete Mitostress experiment. Bars represent the average of the indicated replicates. Each dot in bar graphs represents an independent cell sample. Error bars indicate the standard deviation. Statistical significance was assessed using the two-tailed unpaired Student t test (b, c, k-m) or the one-way ANOVA with Tukey's correction for multiple comparisons (d, f, g). P values are indicated when P<0.05. Source data are provided as a Source Data file.

Figure S2

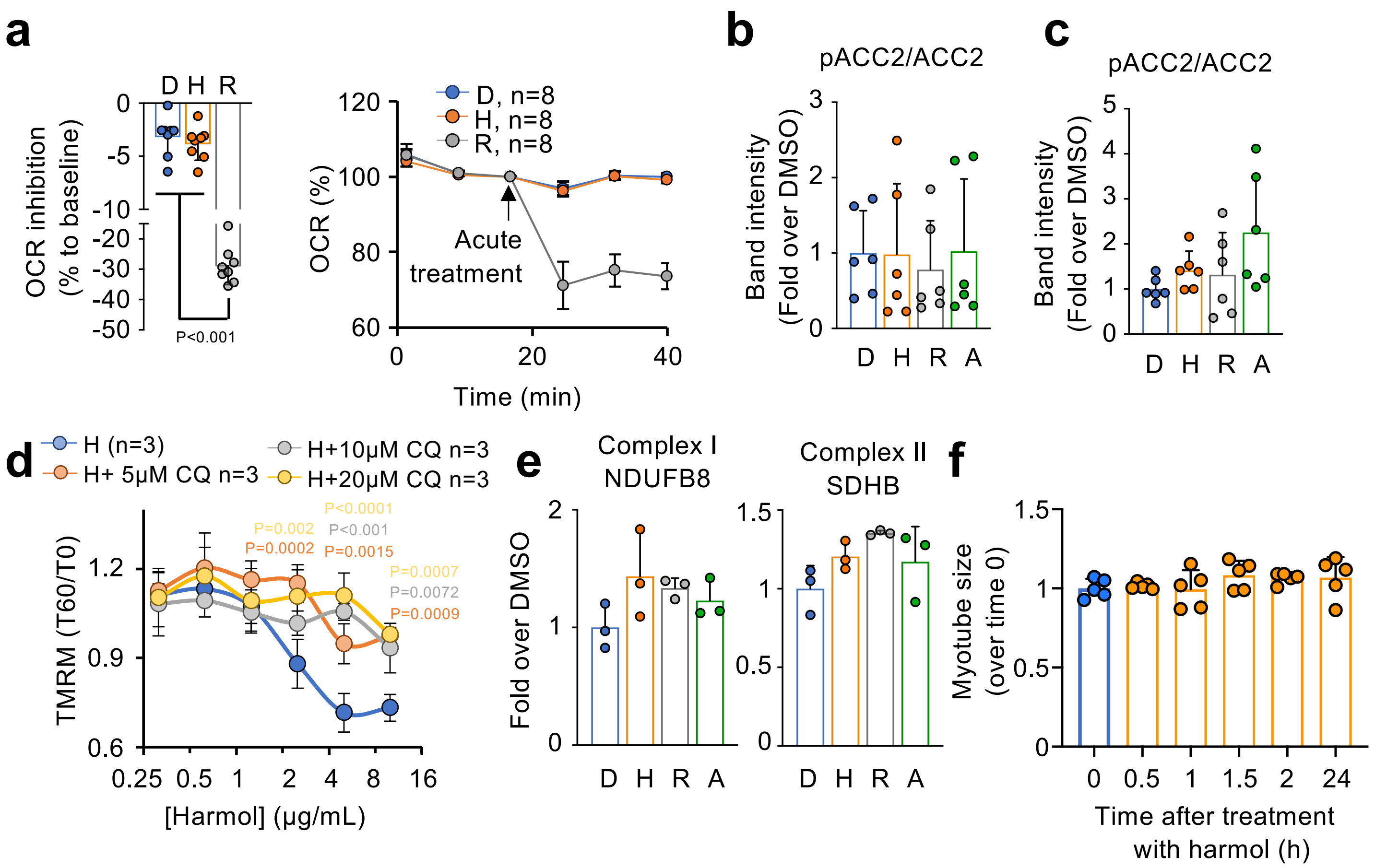


**Supplementary Figure S2. (a)** Differentiated C2C12 myotubes were acutely treated with vehicle (0.1% DMSO, D), 1.3 μg/ml harmol (H) or 30 μM rosiglitazone (R), and oxygen consumption rate (OCR) was determined using Seahorse. A time-course of the OCR after these treatments is shown in the panel to the right. **(b-c)** Quantification of the Western blots against pACC2/ACC2 shown in Figure 2a (b) or against pACC2/ACC2 shown in Figure 2b (c). **(d)** C2C12 myotubes stained with TMRM for 16 hours were treated with harmol alone or together with chloroquine (CQ) at the indicated concentrations for 30 minutes, and TMRM intensity was recorded before (T0) and 60 minutes after (T60) treatment with harmol. **(e)** Quantification of the Westerns blots against the Complex I subunit NDUFB8 or the Complex II subunit SDHB shown in Figure 2f. **(f)** Differentiated C2C12 myotubes were treated with 1.3 μg/ml harmol, and myotube size was measured at the indicated times after treatment. Bars and line-connected dots represent the average of the indicated replicates. Dots in bar graphs (a, b-c, e-f) represent independent cell samples. Error bars represent the standard deviation. Statistical significance was assessed using the one-way ANOVA (a-c, e-f) or the two-way ANOVA (d) with Tukey's correction for multiple comparisons. P values are indicated when P<0.05. Source Data are provided as a Source Data file.

# Figure S3

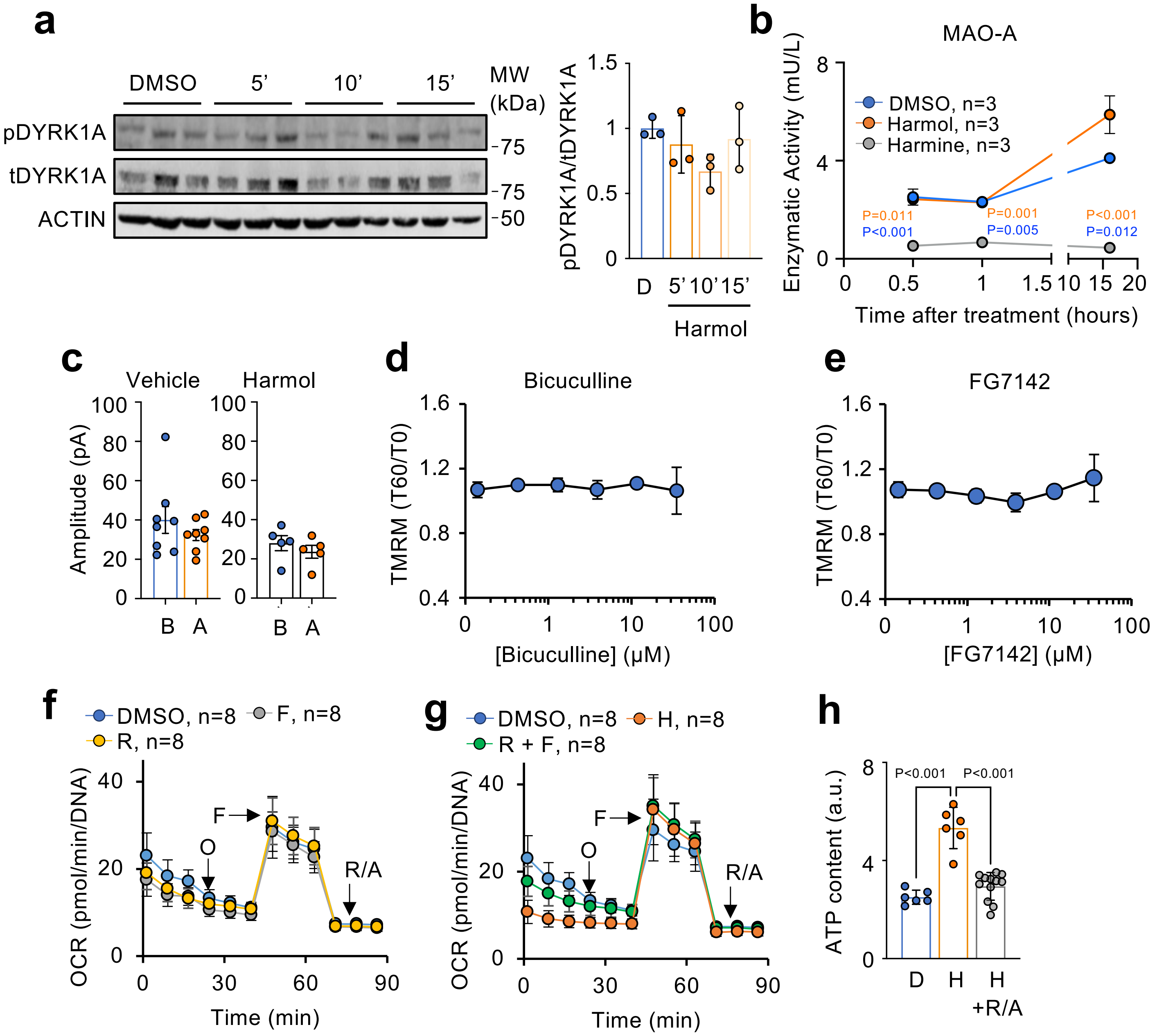
a
DMSO
5'
10'
15'
MW (kDa)
pDYRK1A
tDYRK1A
ACTIN
-75
-75
-50
pDYRK1A/tDYRK1A
D
5'
10'
15'
Harmol
b
MAO-A
DMSO, n=3
Harmol, n=3
Harmine, n=3
Enzymatic Activity (mU/L)
P=0.011
P<0.001
P=0.001
P=0.005
P<0.001
P=0.012
Time after treatment (hours)
c
Vehicle
Harmol
Amplitude (pA)
B
A
d
Bicuculline
TMRM (T60/T0)
[Bicuculline] (μM)
e
FG7142
TMRM (T60/T0)
[FG7142] (μM)
f
DMSO, n=8
F, n=8
R, n=8
OCR (pmol/min/DNA)
O
F
R/A
Time (min)
g
DMSO, n=8
H, n=8
R + F, n=8
OCR (pmol/min/DNA)
O
F
R/A
Time (min)
h
ATP content (a.u.)
P<0.001
P<0.001
D
H
H +R/A

**Supplementary Figure S3. (a)** Differentiated C2C12 myotubes were treated with 0.1% DMSO (D) or 1.3 μg/ml harmol for the indicated times, and whole protein lysates were analyzed by Western blot for he indicated proteins. Band quantification is shown to the right. **(b)** Differentiated C2C12 myotubes were treated with 0.1% DMSO, 1.3 μg/ml harmol or 1.3 μg/ml harmine, and specific MAO-A activity was measured at the indicate times. **(c)** Amplitude of spontaneous inhibitory currents (sIPSCs) events measured in mouse hippocampal slices (n=8 from 3 different animals) before (B) and after treatment (A) with 1.3 μg/ml harmol. **(d-e)** Differentiated C2C12 myotubes (n=3) were treated for 30 minutes with the indicated concentrations of the GABAAR antagonist bicuculline (d) or the GABAAR inverse agonist FG7142 (e), and mitochondrial potential was measured by TMRM fluorescence. **(f-g)** Oxygen consumption rates (OCR) in differentiated C2C12 myotubes treated for 16 hours with rasagilin (R, 2 μM), FG7142 (F, 1 μM) the combination of R + F, or harmol (H, 1.3 μg/ml) following the sequential addition of oligomycin A (Oligo), FCCP and Rotenone/Antimycin A (R/A) at the indicated times (Mitostress Seahorse experiment). **(h)** ATP production was measured in C2C12 myotubes after treatment with 0.1% DMSO (D) or 1.3 μg/ml harmol (H) for 16 hours, followed by treatment with 1 μM Rotenone and 1 μM Antimycin A (R/A) for 1 hour where indicated (grey bar). Bars and line-connected dots represent the average of the indicated replicates. Dots in bar graphs (a, c, h) represent independent cell samples.Error bars represent the standard deviation. Statistical significance was assessed using the one-way ANOVA (a, h) or the two-way ANOVA (b) with Tukey's correction for multiple comparisons; or the two-tailed unpaired Student t test (c). P values are indicated when $P<0.05$. Source data are provided as a Source Data file.

Figure S4

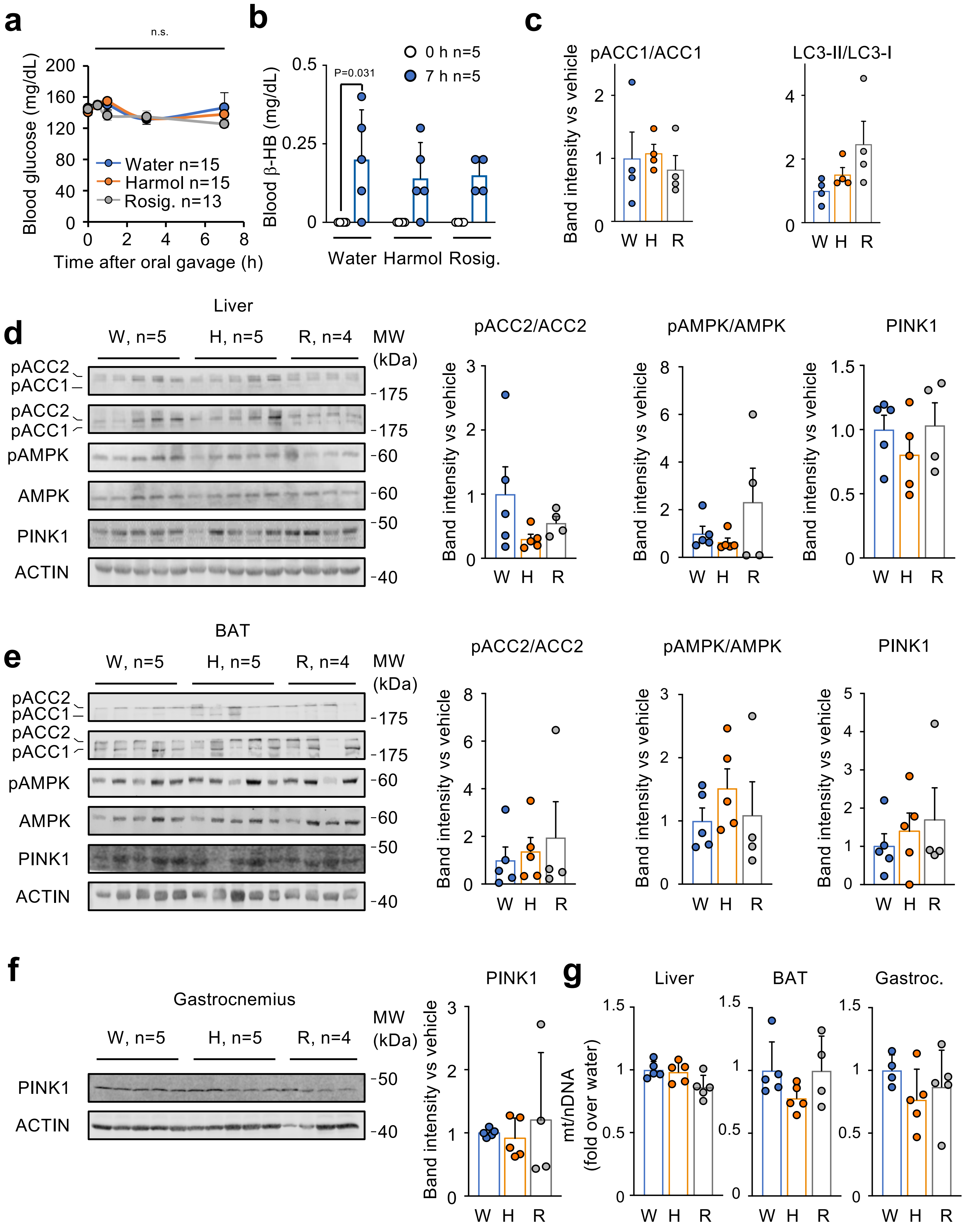

a
n.s.
Blood glucose (mg/dL)
Time after oral gavage (h)
Water n=15
Harmol n=15
Rosig. n=13
b
P=0.031
0 h n=5
7 h n=5
Blood β-HB (mg/dL)
Water Harmol Rosig.
c
pACC1/ACC1
LC3-II/LC3-I
Band intensity vs vehicle
W H R
d
Liver
W, n=5
H, n=5
R, n=4
MW (kDa)
pACC2
pACC1
pAMPK
AMPK
PINK1
ACTIN
-175
-60
-50
-40
pACC2/ACC2
pAMPK/AMPK
PINK1
e
BAT
f
Gastrocnemius
g
Liver
BAT
Gastroc.
mt/nDNA (fold over water)

**Supplementary Figure S4. (a, b)** Blood glucose levels at the indicated times (a) or β-hydroxybutyrate (β-HB) levels 7 hours (b) after oral gavage administration of water, 100 mg/kg harmol or 10 mg/kg rosiglitazone. **(c)** Quantification of the Western blots shown in Figure 4c of the indicated proteins in brain 7 hours after oral gavage administration of vehicle (water, V), 100 mg/kg harmol (H) or 10 mg/kg rosiglitazone (R). **(d-f)** Western blots of the indicated proteins in liver (d), brown adipose tissue (BAT, e) or gastrocnemius muscle (f) 3 hours after oral gavage administration of vehicle (water, W), 100 mg/kg harmol (H) or 10 mg/kg rosiglitazone (R). Quantifications of the indicated proteins are shown in the bar graphs to the right (n=5 for vehicle and harmol, and n=4 for rosiglitazone for each condition). **(g)** Quantitative PCR experiment measuring the ratio between mitochondrial and nuclear DNA (mt/nDNA) in the indicated tissues 3 hours after treatment from the same mice as shown in (c-f). Bars and line-connected dots represent the average of the indicated replicates. Dots in bar graphs (b-g) represent samples or measures from independent animals. Each lane in Western blots (d-f) represents samples from independent animals.Error bars represent the standard error of the mean. Statistical significance was assessed using the two-way ANOVA (a) or the one-way ANOVA (c-g) with Tukey's correction for multiple comparisons; or the paired two-tailed Student t test (b). P values are indicated when $P<0.05$. Source data are provided as a Source Data file.

# Figure S5

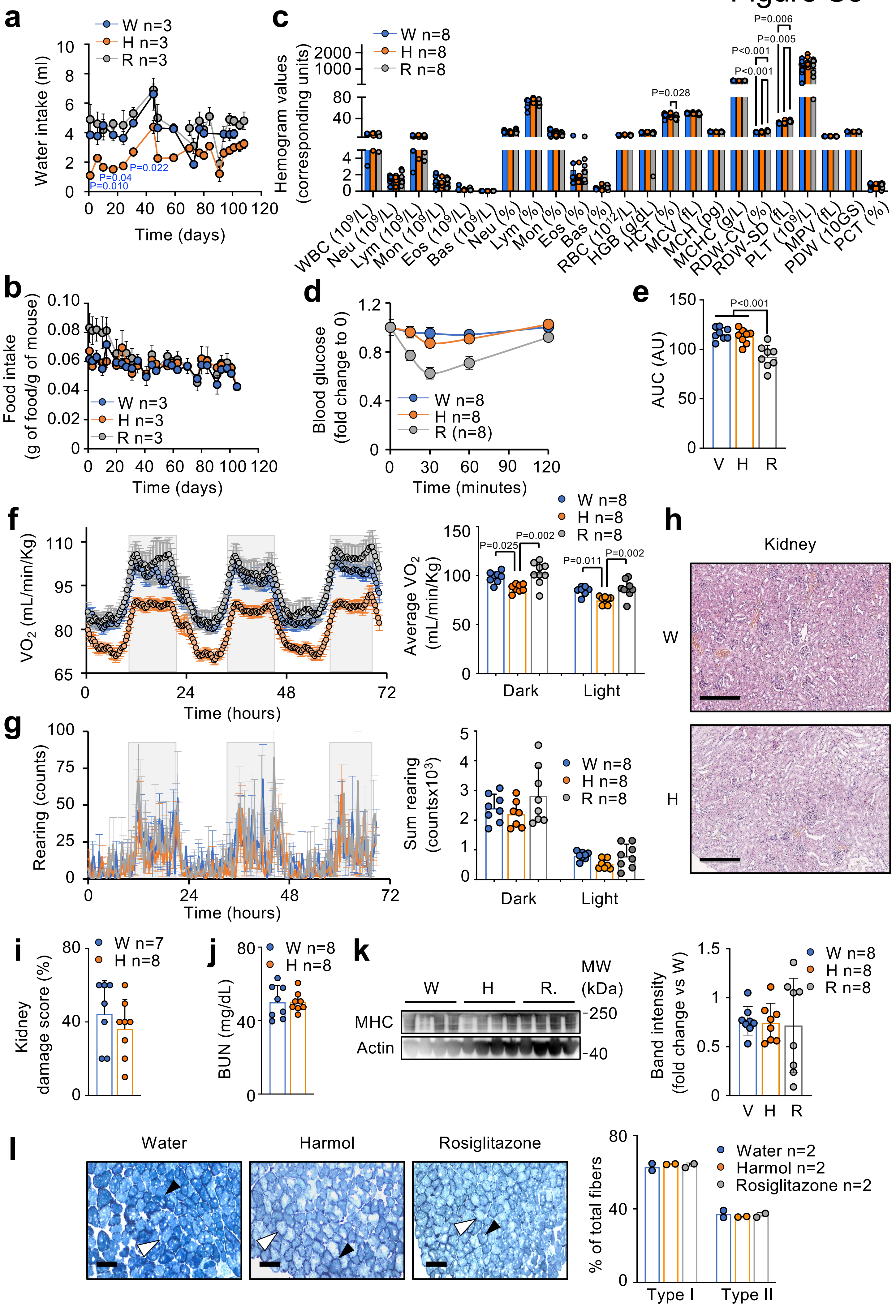

a
W n=3
H n=3
R n=3
Water intake (ml)
Time (days)
P=0.022
P=0.04
P=0.010
b
Food intake (g of food/g of mouse)
Time (days)
c
W n=8
H n=8
R n=8
Hemogram values (corresponding units)
P=0.028
P<0.001
P<0.001
P=0.005
P=0.006
WBC (10^9/L)
Neu (10^9/L)
Lym (10^9/L)
Mon (10^9/L)
Eos (10^9/L)
Bas (10^9/L)
Neu (%)
Lym (%)
Mon (%)
Eos (%)
Bas (%)
RBC (10^12/L)
HGB (g/dL)
HCT (%)
MCV (fL)
MCH (pg)
MCHC (g/L)
RDW-CV (%)
RDW-SD (fL)
PLT (10^9/L)
MPV (fL)
PDW (10GS)
PCT (%)
d
Blood glucose (fold change to 0)
W n=8
H n=8
R (n=8)
Time (minutes)
e
AUC (AU)
P<0.001
V H R
f
VO2 (mL/min/Kg)
Time (hours)
Average VO2 (mL/min/Kg)
P=0.025
P=0.002
P=0.011
P=0.002
Dark
Light
g
Rearing (counts)
Time (hours)
Sum rearing (countsx10^3)
h
Kidney
W
H
i
Kidney damage score (%)
W n=7
H n=8
j
BUN (mg/dL)
W n=8
H n=8
k
W H R.
MW (kDa)
MHC
Actin
-250
-40
Band intensity (fold change vs W)
V H R
l
Water
Harmol
Rosiglitazone
% of total fibers
Water n=2
Harmol n=2
Rosiglitazone n=2
Type I
Type II

**Supplementary Figure S5. (a, b)** Water (a) and food (b) intake of the same mice shown in Figure 5 during the treatment with vehicle (water, W), 100 mg/kg harmol (H) or 10 mg/kg rosiglitazone (R). **(c)** Hemogram values obtained from the blood of the same mice described in Figure 5 at the time of sacrifice. **(d, e)** Insulin tolerance test (d) and quantification of the area under the curve (AUC) in the same mice described in Figure 5 at the end of the treatment with the indicated compounds. **(f, g)** $VO_2$ (f) and rearing activity counts (g) from the indirect calorimetry study with the same mice from Figure 5. Average values (f) or total rearing counts (g), divided in dark and night periods, are shown in the panels to the right. **(h)** Representative images of hematoxilin and eosin staining of kidney sections of the same mice described in Figure 5. Size bar=500μm **(i)** Quantification of the histological score using the kidney sections stained with hematoxilin and eosin shown in (h). **(j)** Blood urea nitrogen (BUN) measured at sacrifice in the mice described in Figure 5. **(k)** Western blot against total myosin heavy chain (MHC) in protein extracts from soleus muscle of obese pre-diabetic male mice treated with the indicated treatments. Band quantification is shown in the panel to the right. **(l)** Fiber typing in soleus muscle cryosections stained with NADH solution from a subset of the same mice shown in Figure 5. Black arrows: type I fibers. White arrows: type II fibers. Size bar=100μm. Bars and line-connected dots represent the average of the indicated replicates. Dots in bar graphs (c, e-g, i-l) represent samples or measures from independent animals. Error bars represent the standard error of the mean. Statistical significance was assessed using the two-way ANOVA (a, b, d); the one-way ANOVA (c, e-g, k-l) with Tukey's correction for multiple comparisons; or the unpaired two-tailed Student's t test (i, j). P values are indicated when $P<0.05$. Source data are provided as a Source Data file.

# Figure S6

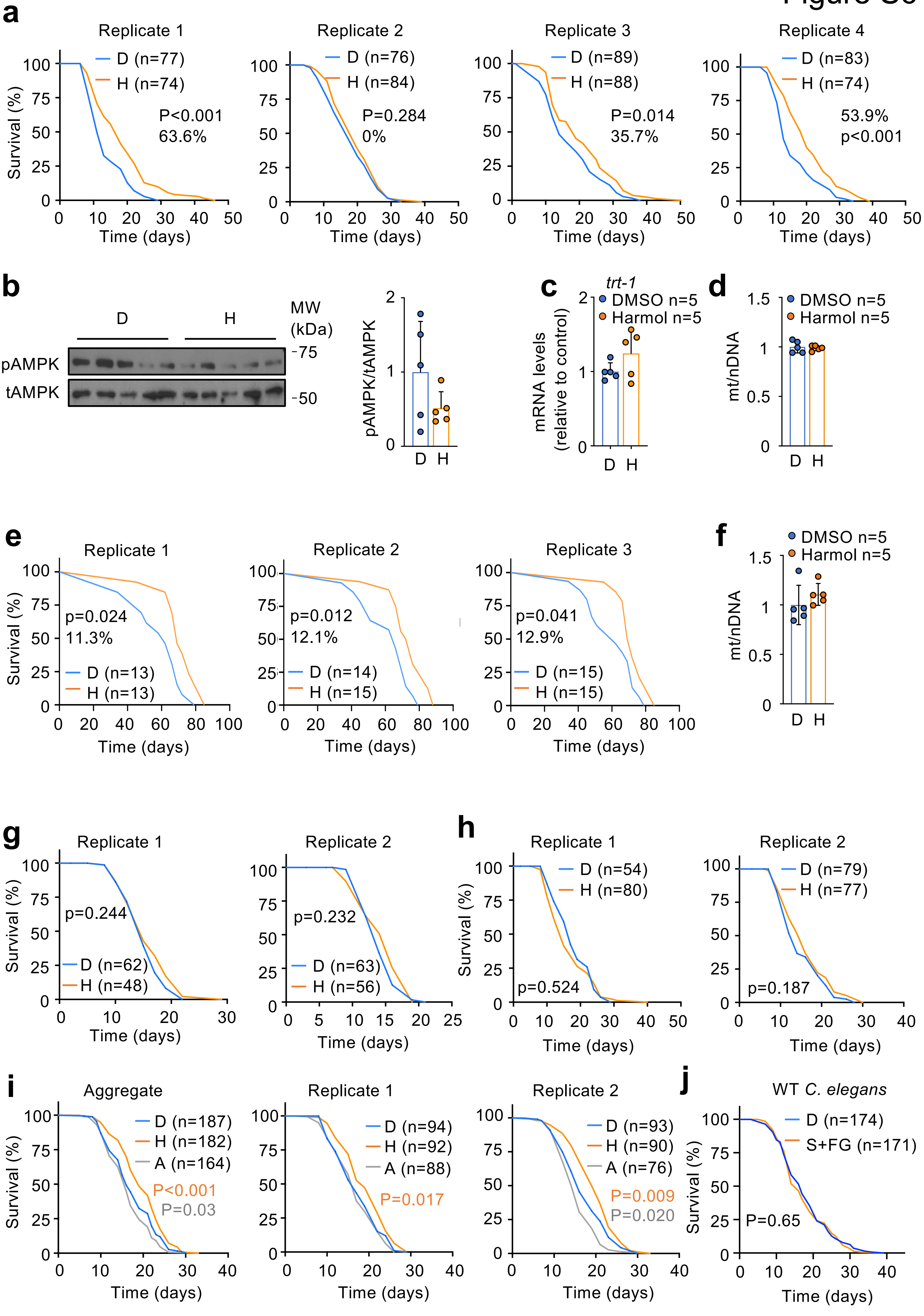
a
Replicate 1
D (n=77)
H (n=74)
P<0.001
63.6%
Replicate 2
D (n=76)
H (n=84)
P=0.284
0%
Replicate 3
D (n=89)
H (n=88)
P=0.014
35.7%
Replicate 4
D (n=83)
H (n=74)
53.9%
p<0.001
Survival (%)
Time (days)
b
D
H
MW (kDa)
pAMPK
tAMPK
-75
-50
pAMPK/tAMPK
c
trt-1
DMSO n=5
Harmol n=5
mRNA levels (relative to control)
d
DMSO n=5
Harmol n=5
mt/nDNA
e
Replicate 1
p=0.024
11.3%
D (n=13)
H (n=13)
Replicate 2
p=0.012
12.1%
D (n=14)
H (n=15)
Replicate 3
p=0.041
12.9%
D (n=15)
H (n=15)
f
DMSO n=5
Harmol n=5
mt/nDNA
g
Replicate 1
p=0.244
D (n=62)
H (n=48)
Replicate 2
p=0.232
D (n=63)
H (n=56)
h
Replicate 1
D (n=54)
H (n=80)
p=0.524
Replicate 2
D (n=79)
H (n=77)
p=0.187
i
Aggregate
D (n=187)
H (n=182)
A (n=164)
P<0.001
P=0.03
Replicate 1
D (n=94)
H (n=92)
A (n=88)
P=0.017
Replicate 2
D (n=93)
H (n=90)
A (n=76)
P=0.009
P=0.020
j
WT C. elegans
D (n=174)
S+FG (n=171)
P=0.65

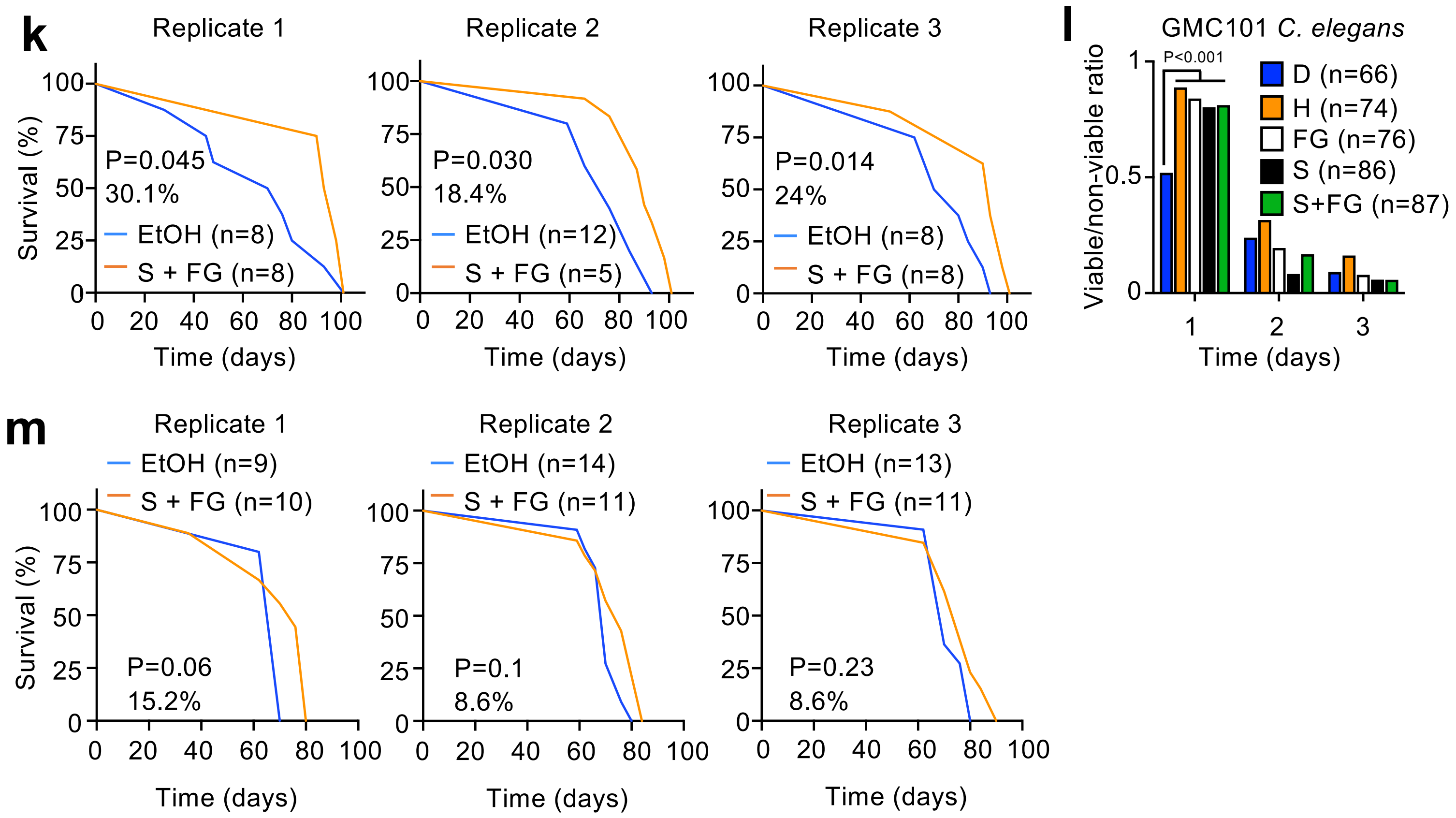


**Supplementary Figure S6. Treatment with harmol extends lifespan and healthspan in worms and flies. (a)** Individual replicates of the Kaplan-Meier survival curves of WT *C. elegans* worms treated with vehicle (DMSO, D) or 15 µg/ml harmol (H). **(b-d)** WT *C. elegans* worms were treated with vehicle (DMSO, D) or 15 µg/ml harmol (H) from birth to the end of their development, and analyzed for the indicated proteins by Western blot (b, band quantification is shown in the graph to the right); for the expression of the *trt-1* gene by qPCR (c); or for the ratio of mitochondrial vs nuclear DNA (mt/nDNA, d). **(e)** Individual replicates of the Kaplan-Meier survival curves of WT *D. melanogaster* flies treated with vehicle (DMSO, D) or with 25 µg/ml harmol (H). **(f)** WT *D. melanogaster* flies were treated with vehicle (DMSO, D) or 25 µg/ml harmol (H) for 2 weeks, and were then analyzed for the ratio of mitochondrial vs nuclear DNA (mt/nDNA). **(g-k)** Individual replicates of the Kaplan-Meier survival curves of **(g)** AMPK homologue *aak-2* mutant worms treated with vehicle (DMSO, D) or 15 µg/ml harmol (H); **(h)** *C. elegans* worms mutant for the GABAAR homologue *unc-49* treated with vehicle (DMSO, D) or 15 µg/ml harmol (H); **(i)** MAO homologues *amx-1/2/3* triple mutant worms treated with vehicle (DMSO, D), 15 µg/ml harmol (H), or with 1mM AICAR (A); **(j)** WT *C. elegans* worms treated with vehicle (DMSO, D) or with a combination of 0.45 µg/ml selegiline and 6 µg/ml FG7142; **(k)** WT *D. melanogaster* flies treated with vehicle (ethanol, EtOH) or with a combination of 0.75 µg/ml selegiline (S) and 10 µg/ml FG7142 (F). **(l)** Viable/non-viable ratio of GMC101 mutant *C. elegans* worms 1, 2 or 3 days after treatment with vehicle (DMSO, D), 15µg/ml harmol (H) or with a combination of 0.45 µg/ml selegiline (S) and 6 µg/ml FG7142 (F); **(m)** Individual replicates of the Kaplan-Meier survival curves of *para*$^{bss1}$ mutant *D. melanogaster* flies treated with vehicle (ethanol, EtOH) or with a combination of 0.75 µg/ml selegiline (S) and 10 µg/ml FG7142 (F). Dots in bar graphs (b-d, f) represent samples or measures from independent animals. Statistical significance was calculated using the logrank test (a, e, g-k, m); the unpaired two-tailed Student t test (b-d, f); or the Fisher's exact test (l). In Figure S6i, color codes indicate the precise curve being compared with vehicle control. Source data are provided as a Source Data file.

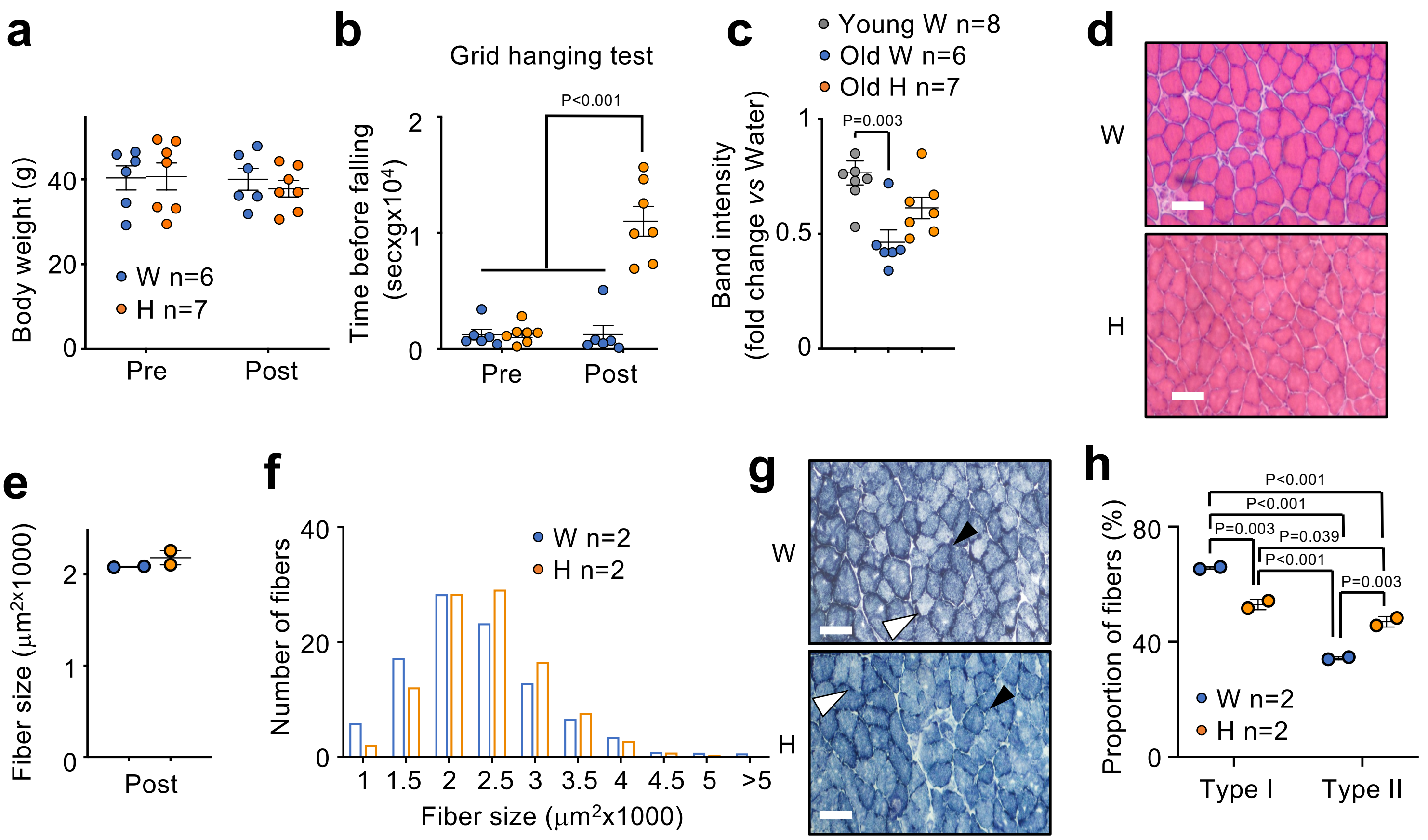


**Supplementary Figure S7. Treatment with harmol delays aging and reduces frailty in old mice. (a, b)** Changes in total body weight (a); or in hanging endurance in the grid hanging test, normalized by mouse body weight (b), in 2-year-old mice before (Pre) and after (Post) treatment with 100 mg/kg harmol in the drinking water for 2 months. **(c)** Quantification of total myosin heavy chain (MHC) WB in protein extracts from soleus muscle of the 7 month-old water-treated mice on HFD shown in Figure 5 (Young W); 2 year-old mice treated with water (Old W); or 2-year old mice treated with 100 mg/kg harmol (Old H). **(d-f)** Cross-sectional area of soleus muscle fibers of a subset of the aged male mice shown in (a), treated with water (vehicle, W) or 100 mg/kg harmol (H), was measured in cryosections stained with H&E (representative examples shown in d) and quantified as gross fiber size (e) or fiber size distribution (f). Size bar=100μm. **(g)** Fiber typing in soleus muscle cryosections stained with NADH solution from mice treated with water (vehicle, W) or 100 mg/kg harmol (H). Black arrows: type I fibers. White arrows: type II fibers. Size bar=100μm. **(h)** Quantification of the proportion of fibers shown in (g). Dots represent samples or measures from independent animals. Statistical significance was calculated using the two-way ANOVA test with Tukey's correction for multiple comparisons (a, b, h); the one-way ANOVA test with Tukey's correction for multiple comparisons (c); or the unpaired two-tailed Student t test (e). P values are indicated when P<0.05. Source data are provided as a Source Data file.

**Supplemental Table 1. PAMPA-BBB-based determination of permeability to the blood-brain barrier.**

| | Harmol | Harmine | Norharmane | Serotonin |
|---|---|---|---|---|
| **LogPe** | -4.01 | -4.83 | -3.94 | -4.72 |
| **R(%)** | 54.50 | 92.29 | 71.64 | 4.80 |
| **Ca(t)/Cd(0) (%)** | 24.64 | 6.17 | 26.29 | 7.72 |

**Supplementary Table 2.** LC-MS/MS conditions for determination of harmol and harmine-$d_3$.

| Compound | Retention time (min) | Precursor ion | Cone voltage (V) | Product ion | Collision energy (eV) |
|---|---|---|---|---|---|
| Harmol | 4.43 | 199.2 | 30 | 77.0 | 40 |
| | | | | 103.0 | 35 |
| | | | | 131.0 | 35 |
| | | | | 171.0 | 30 |
| | | | | 184.1 | 30 |
| Harmine-$d_3$ | 6.47 | 216.1 | 30 | 144.0 | 35 |
| | | | | 169.0 | 45 |
| | | | | 170.0 | 30 |
| | | | | 198.0 | 25 |
