# Supplementary material for: Peripheral modulation of antidepressant targets MAO-B and GABAAR by harmol induces mitohormesis and delays aging in preclinical models
Source: Nat Commun. 2023 May 15;14:2779. doi: 10.1038/s41467-023-38410-y (PMC10185515; doi:10.1038/s41467-023-38410-y)
Supplement: Supplementary file 3 — Description of Additional Supplementary Files [file 41467_2023_38410_MOESM3_ESM.pdf]

Description of Additional Supplementary Files

File Name: Supplementary Data 1
Description: List of compounds tested in the screening shown in Figure 1 and Supplementary Figure 1.

File Name: Supplementary Data 2
Description: Spermidine quantification in C2C12 myotubes (Figure 3c) or liver (Figure 5r) of cells or mice treated with harmol.

File Name: Supplementary Data 3
Description: Harmol quantification in plasma, liver or brain from mice treated with harmol at eh indicated times after harmol administration, complementing Figure 4a-b.

File Name: Supplementary Data 4
Description: Survival data of *C. elegans* worms or *D. melanogaster* flies under the indicated treatments, complementing Figure 6.
