# Supplementary material for: Peripheral modulation of antidepressant targets MAO-B and GABAAR by harmol induces mitohormesis and delays aging in preclinical models
Source: Nat Commun. 2023 May 15;14:2779. doi: 10.1038/s41467-023-38410-y (PMC10185515; doi:10.1038/s41467-023-38410-y)
Supplement: Supplementary file 5 — Reporting Summary [file 41467_2023_38410_MOESM5_ESM.pdf]

nature portfolio

Corresponding author(s): Pablo J. Fernandez-Marcos

Last updated by author(s): 27-03-2023

Reporting Summary

Nature Portfolio wishes to improve the reproducibility of the work that we publish. This form provides structure for consistency and transparency in reporting. For further information on Nature Portfolio policies, see our Editorial Policies and the Editorial Policy Checklist.

Statistics

For all statistical analyses, confirm that the following items are present in the figure legend, table legend, main text, or Methods section.

n/a	Confirmed	
☐	☒	The exact sample size (*n*) for each experimental group/condition, given as a discrete number and unit of measurement
☐	☒	A statement on whether measurements were taken from distinct samples or whether the same sample was measured repeatedly
☐	☒	The statistical test(s) used AND whether they are one- or two-sided<br>*Only common tests should be described solely by name; describe more complex techniques in the Methods section.*
☐	☒	A description of all covariates tested
☐	☒	A description of any assumptions or corrections, such as tests of normality and adjustment for multiple comparisons
☐	☒	A full description of the statistical parameters including central tendency (e.g. means) or other basic estimates (e.g. regression coefficient) AND variation (e.g. standard deviation) or associated estimates of uncertainty (e.g. confidence intervals)
☐	☒	For null hypothesis testing, the test statistic (e.g. *F*, *t*, *r*) with confidence intervals, effect sizes, degrees of freedom and *P* value noted<br>*Give P values as exact values whenever suitable.*
☒	☐	For Bayesian analysis, information on the choice of priors and Markov chain Monte Carlo settings
☒	☐	For hierarchical and complex designs, identification of the appropriate level for tests and full reporting of outcomes
☐	☒	Estimates of effect sizes (e.g. Cohen's *d*, Pearson's *r*), indicating how they were calculated

Our web collection on statistics for biologists contains articles on many of the points above.

Software and code

Policy information about availability of computer code

Data collection	Mitochondrial membrane potential was determined using the Opera High Content Screening System (PerkinElmer Opera EvoShell 2.0.0.12199).<br>Seahorse data was collected using an Agilent Seahorse XFe analyzer with the Wave 2.4.3 Seahorse software.<br>qPCR data was collected using an an ABI PRISM 7900 thermocycler with the Sequence Detection Systems SDS 2.4.1 software (Applied Biosystems).<br>LC-MS/MS data were acquired and processed using MassLynx 4.2 software (Waters Corp, Manchester, UK) and TargetLynx application (Waters Corp, Manchester, UK).<br>Body composition (fat and lean content) was collected by dual energy X-ray Absorptiometry (DEXA) using a Lunar PIXImus Densitometer (GE Medical Systems).

Data analysis	Analysis of confocal images was performed using Definens Developer XD v2.5 (Definiens) and Acapella 2.6 (Perkin Elmer).<br>Western blot band quantification, muscle morphometry analysis and worm viability analysis were conducted using ImageJ 1.53n.<br>Data from Seahorse experiments were analyzed using Wave 2.4.3 Seahorse software.<br>qPCR data was analyzed with the Sequence Detection Systems SDS 2.4.1 software (Applied Biosystems).<br>Electrophysiological readings were digitized with 1440A Digidata and analyzed using pCLAMP and MiniAnalysis software.<br>LC-MS/MS data were acquired and processed using MassLynx 4.2 software (Waters Corp, Manchester, UK) and TargetLynx application (Waters Corp, Manchester, UK).<br>Body composition (fat and lean content) was analyzed with the PIXImus II Series Densitometers software version 1.46.007 (GEHC).<br>Statistical analysis of data was performed using GraphPad Prism 9 for MacOS.

For manuscripts utilizing custom algorithms or software that are central to the research but not yet described in published literature, software must be made available to editors and reviewers. We strongly encourage code deposition in a community repository (e.g. GitHub). See the Nature Portfolio guidelines for submitting code & software for further information.

Data

Policy information about availability of data

All manuscripts must include a data availability statement. This statement should provide the following information, where applicable:

- Accession codes, unique identifiers, or web links for publicly available datasets
- A description of any restrictions on data availability
- For clinical datasets or third party data, please ensure that the statement adheres to our policy

Mass spectrometry data can be found at www.ebi.ac.uk/metabolights/MTBLS7533, code MTBLS7533. All other data is available in the manuscript, in the Supplementary Figures, and in the Source Data files provided with this paper.

Human research participants

Policy information about studies involving human research participants and Sex and Gender in Research.

Reporting on sex and gender	N.a.
Population characteristics	N.a.
Recruitment	N.a.
Ethics oversight	N.a.

Note that full information on the approval of the study protocol must also be provided in the manuscript.

Field-specific reporting

Please select the one below that is the best fit for your research. If you are not sure, read the appropriate sections before making your selection.

☒ Life sciences ☐ Behavioural & social sciences ☐ Ecological, evolutionary & environmental sciences

For a reference copy of the document with all sections, see nature.com/documents/nr-reporting-summary-flat.pdf

Life sciences study design

All studies must disclose on these points even when the disclosure is negative.

Sample size	Previous reports on similar mouse (Sierra-Ramirez et al., 2021; Arc-Chagnaud et al., 2021), fly (Tapia et al., 2021) and worm (McIntyre et al., 2021) projects yielded statistically significant results with similar sample sizes.
Data exclusions	No data were excluded from the analyses.
Replication	At least 3 independent replicates for each parameter were analyzed. Some results (identification of positive hits by TMRM and Seahorse - Figure 1; colocalization and ATP experiments in Figure 2; a large part of the metabolic experiments in Figure 5) were reproduced once more, although only the most complete sets of results are presented in this article. We only present results for which all technically correct attempts at replication were successful.
Randomization	In the experiments including groups, group allocation was random, insuring homogeneous age, sex and metabolic status for all groups before the interventions.
Blinding	For mouse experiments, histological analysis were performed in a blinded fashion. For other molecular analyses of cell, mouse or invertebrate samples, blinding was not performed because all samples were treated equally and measurements were obtained in an automated, non-

subjective fashion.

Reporting for specific materials, systems and methods

We require information from authors about some types of materials, experimental systems and methods used in many studies. Here, indicate whether each material, system or method listed is relevant to your study. If you are not sure if a list item applies to your research, read the appropriate section before selecting a response.

Materials & experimental systems

n/a	Involved in the study
☐	☒ Antibodies
☐	☒ Eukaryotic cell lines
☒	☐ Palaeontology and archaeology
☐	☒ Animals and other organisms
☒	☐ Clinical data
☒	☐ Dual use research of concern

Methods

n/a	Involved in the study
☒	☐ ChIP-seq
☒	☐ Flow cytometry
☒	☐ MRI-based neuroimaging

Antibodies

Antibodies used

For Western blots, primary antibodies against mouse phospho-AMPKα (Thr172) (pAMPK, 1:250 – 40H9), AMPKα (1:1000 – 2532S), LC3 (1:500 – 2775S), phospho-Acetyl-CoA Carboxylase (Ser79) (pACC) (1:500 – 3661S), ACC (1:1000 – 3676S), PINK1 (1:500 – 6946T) from Cell Signaling; TFAM (1:500 – ab47517), Total OXPHOS Rodent WB Antibody Cocktail (1:1000 – ab110413), Adiponectin (1:500 – ab22554) from Abcam; Transferrin (1:500 – sc-373785) and Total DYRK1A (1:1000 - sc-100376) from Santa Cruz Biotechnology; c-tubulin (1:20,000, GTU-88) or Actin (1:20000 - A1978) from Sigma; Anti-Myosin heavy chain, sarcomere (1:1000 - AB_2147781) from Developmental Studies Hybridoma Bank; and phospho-DYRK1A (1:1000 – 15728122) from Invitrogen; were used. Primary antibodies were incubated with anti-mouse (1:10000 IRDye 800CW, 926-32210, lot number D01110-03) or anti-rabbit (1:10000 IRDye 680RD, 926-68071, lot number D00819-05) secondary antibodies from Li-COR in a mixture of 5% non-fat milk dissolved in TBS-T for 1 hour at room temperature.

Validation

The expression patterns and/or subcellular localization of all the proteins analyzed by using comercial antibodies have been previously validated by the manufacturers and widely used in researches. Methods of validation such as immunofluorescences and western blots and references have been published for all antibodies and are all present into manufacturer dedicated website page of each indicated product.
Mouse phospho-AMPKα (Thr172) (pAMPK, 40H9) was validated by Western blot by manufacturer against the cells used in this study (C2C12): https://www.cellsignal.com/products/primary-antibodies/phospho-ampka-thr172-40h9-rabbit-mab/2535?site-search-type=Products&N=4294956287&Ntt=phospho-ampk%CE%B1+%28thr172%29+%2840h9%29+rabbit+mab+%232535&fromPage=plp&_requestid=1211386)
AMPKα, 2532S was validated by Western blot by manufacturer against the cells used in this study (C2C12): https://www.cellsignal.com/products/primary-antibodies/ampka-antibody/2532?site-search-type=Products&N=4294956287&Ntt=ampk%CE%B1++2532s&fromPage=plp&_requestid=1211446
LC3, 2775S, was validated by Western blot by manufacturer, and cross reactivity against mouse was indicated: https://www.cellsignal.com/products/primary-antibodies/lc3b-antibody/2775?site-search-type=Products&N=4294956287&Ntt=lc3+2775s&fromPage=plp&_requestid=1211687
phospho-Acetyl-CoA Carboxylase (Ser79) (pACC, 3661S) was validated by Western blot by manufacturer, and cross reactivity against mouse was indicated: https://www.cellsignal.com/products/primary-antibodies/phospho-acetyl-coa-carboxylase-ser79-antibody/3661?site-search-type=Products&N=4294956287&Ntt=ser79%29+%28pacc%29++3661s&fromPage=plp&_requestid=1212192
ACC, 3676S was validated by Western blot against mouse cell lines by manufacturer: https://www.cellsignal.com/products/primary-antibodies/acetyl-coa-carboxylase-c83b10-rabbit-mab/3676?site-search-type=Products&N=4294956287&Ntt=acc+3676s&fromPage=plp&_requestid=1212475
PINK1, 6946T was validated by Western blot by manufacturer: https://www.cellsignal.com/products/primary-antibodies/pink1-d8g3-rabbit-mab/6946?site-search-type=Products&N=4294956287&Ntt=pink1+6946t&fromPage=plp&_requestid=1212869
TFAM, ab47517 was validated by Western blot against mouse tissues by manufacturer: https://www.abcam.com/products/primary-antibodies/mttfa-antibody-mitochondrial-marker-ab47517.html
Total OXPHOS Rodent WB Antibody Cocktail, ab110413 was validated by Western blot against mouse tissues by manufacturer: https://www.abcam.com/products/panels/total-oxphos-rodent-wb-antibody-cocktail-ab110413.html
Adiponectin, ab22554 was validated by Western blot by manufacturer, and cross reactivity against mouse was indicated: https://www.abcam.com/products/primary-antibodies/adiponectin-antibody-19f1-ab22554.html
Transferrin, sc-373785 was validated by Western blot against mouse tissues by manufacturer: https://www.scbt.com/es/p/transferrin-antibody-f-8
Total DYRK1A, sc-100376 was validated by Western blot against mouse cell lines by manufacturer: https://www.scbt.com/p/dyrk1a-antibody-rr-7?requestFrom=search
Tubulin, GTU-88 was validated by Western blot by manufacturer: https://www.sigmaaldrich.com/ES/es/product/sigma/t6557?gclid=CjwKCAjwzuqgBhAcEiwAdj5dRk5Ybe3RBeoXHf6kdM8ZzEqU7eB_VC9wHdOi3bpSC2Omh4q4DovJfxoC0dMQAvD_BwE&gclsrc=aw.ds
Actin, A1978 was validated by Western blot against mouse cell lines by manufacturer: https://www.sigmaaldrich.com/ES/es/search/actin-a1978?focus=products&page=1&perpage=30&sort=relevance&term=actin%20a1978&type=product
Anti-Myosin heavy chain, sarcomere, AB_2147781 was validated by Western blot by manufacturer, and cross reactivity against mouse was indicated: https://dshb.biology.uiowa.edu/MF-20

phospho-DYRK1A, 15728122 was validated by Western blot by a prior publication referred by manufacturer, and cross reactivity against mouse was indicated: https://www.thermofisher.com/antibody/product/PA5-64574.html?gclid=CjwKCAjwzuqgBhAcEiwAdj5dRvE_NAKzS-Ypa5PjsslNfC2DZMkrj-JJpZj96KOtfqoiEvFNAkzZ4RoCCPoQAvD_BwE&ef_id=CjwKCAjwzuqgBhAcEiwAdj5dRvE_NAKzS-Ypa5PjsslNfC2DZMkrj-JJpZj96KOtfqoiEvFNAkzZ4RoCCPoQAvD_BwE:G:s&s_kwcid=AL!3652!3!459737518508!!!g!!!10950825775!106531320406&cid=bid_pca_aup_r01_co_cp1359_pjt0000_bid00000_0se_gaw_dy_pur_con

Eukaryotic cell lines

Policy information about cell lines and Sex and Gender in Research

Cell line source(s)	C2C12 murine myoblast cells were obtained from the ATCC
Authentication	None of the cell lines used were autenticated.
Mycoplasma contamination	Cell lines were periodically tested for mycoplasma and did not present positive results.
Commonly misidentified lines (See ICLAC register)	Cells used for this work are not listed in the ISLAC register.

Animals and other research organisms

Policy information about studies involving animals; ARRIVE guidelines recommended for reporting animal research, and Sex and Gender in Research

Laboratory animals	C. elegans strains N2 Bristol, GMC101 (dvIs100 [unc-54p::A-beta-1-42::unc-54 3'-UTR + mtl-2p::GFP]), aak-2(ok524), unc-49(e407) and amx-1(ok659);amx-2(ok1235);amx-3(ok1235) triple mutant were used. For D. melanogaster experiments, hybrid females of a cross between two reference strains, Canton-CS and w1118, both obtained from the Bloomington Drosophila stock center (BDSC, University of Indiana). For the epilepsy model, we used the parabss1 mutant model. For experiments with obses mice, 12-week-old C57BL/6OlaHsd male mice were used. For behavioral studies, 3-4 month-old adult C57BL/6J male mice were used. For frailty studies, 23-month-old C57BL/6J mice (9 males, 4 females) were used.
Wild animals	The study did not involve wild animals.
Reporting on sex	C. elegans experiments were performed with males and females, and no sex determination was performed. D. melanogaster experiments were performed with female only. Mouse expeirments were performed with males only (metabolic and behavioral experiments) or with a mix of males and females (frailty experiments). In this last set of experiments, each treatment group had roughly equal number of males and females: control (n=6; 4 males, 2 females) and harmol-treated (n=7; 5 males, 2 females).
Field-collected samples	This study did not involve data collected from field.
Ethics oversight	For invertebrate studies no ethical approval was required. All mouse experiments were performed according to protocols approved by the CNIO-ISCIII Ethics Committee for Research and Animal Welfare (CEIyBA) (PROEX 161/18); and by the University of Valencia Ethics Committee for Research and Animal Welfare (License reference: A1444079171882).

Note that full information on the approval of the study protocol must also be provided in the manuscript.
